# Time Dependence of Gel Formation in Lyotropic Nematic Liquid Crystals: From Hours to Weeks

**DOI:** 10.3390/gels10040261

**Published:** 2024-04-13

**Authors:** Max Dombrowski, Michael Herbst, Natalie Preisig, Frank Giesselmann, Cosima Stubenrauch

**Affiliations:** Institute of Physical Chemistry, University of Stuttgart, 70569 Stuttgart, Germany

**Keywords:** lyotropic nematic liquid crystals, low-molecular-weight gelator, gel aging, physical gel

## Abstract

The combination of lyotropic liquid crystals (LLCs) and low-molecular-weight gelators (LMWGs) for the formation of lyotropic liquid crystal gels (LLC gels) leads to a versatile and complex material combining properties of both parent systems. We gelled the calamitic nematic N_C_ phases of a binary and ternary system using the LMWG 3,5-bis-(5-hexylcarbamoyl-pentoxy)-benzoic acid hexyl ester (BHPB-6). This binary system consists of the surfactant *N*,*N*-dimethyl-*N*-ethyl-1-hexadecylammonium bromide (CDEAB) and water, whereas the ternary system consists of the surfactant *N*,*N*,*N*-trimethyl-*N*-tetradecylammonium bromide (C_14_TAB), the cosurfactant *n*-decanol, and water. Though containing similar surfactants, the gelled N_C_ phases of the binary and ternary systems show differences in their visual and gel properties. The gelled N_C_ phase of the binary system remains clear for several days after preparation, whereas the gelled N_C_ phase of the ternary system turns turbid within 24 h. We investigated the time evolution of the gel strength with oscillation rheology measurements (a) within the first 24 h and (b) up to two weeks after gel formation. The shape of the fibers was investigated over different time scales with freeze fracture electron microscopy (FFEM). We demonstrate that despite their similarities, the two LLC gels also have distinct differences.

## 1. Introduction

Lyotropic liquid crystal gels (LLC gels) combine the properties of both their parent systems, namely the lyotropic liquid crystal (LLC) and the gel. This results in an anisotropic soft solid which possesses the orientational order of an LLC and the mechanical stability of a gel.

The LLCs studied by us were all surfactant based and were obtained by dissolving a surfactant, and if necessary, a cosurfactant in water. Depending on the concentration of the components and the temperature, various birefringent liquid crystalline phases such as the lamellar L_α_, hexagonal H_1_, discotic nematic N_D_, and calamitic nematic N_C_ can form [1]. The lyotropic nematic liquid crystalline phases are rare compared to the L_α_ and H_1_ phases and most systems require a cosurfactant to form them [2,3].

The gels are formed using a low-molecular-weight gelator (LMWG) which forms a gel using non-covalent interactions such as hydrogen bonding, π stacking, and van der Waals interactions [4,5,6,7]. The gel formation is reversible with heat being the primary trigger for the gelation and dissolution of the gel molecules. As regards LMWGs, one must differentiate between hydrogelators, which are gel aqueous solutions, and organogelators, which are gel organic solvents [8,9,10,11]. This classification, however, is not applicable in our case where the liquid to be gelled is neither an aqueous solution nor an organic solvent. What we are aiming at is to gel complex fluids in general [12,13,14,15,16,17,18,19,20,21,22,23] and nematic lyotropic liquid crystals in particular [22,23]. The gelation of lyotropic liquid crystals can be influenced by interactions between the surfactants composing the lyotropic liquid crystals and the gelator molecules. In this case, the presence of the gelator can cause a shift in the phase boundaries of the lyotropic liquid crystal leading to a phase transition or to a destruction of the lyotropic liquid crystal [12,14,15,21]. If no significant interactions occur between the surfactant and the gelator, the gel network and the lyotropic liquid crystal may form independently of each other, a phenomenon called orthogonal self-assembly [12,13,16,17,24,25]. For the present and the previous study [23], we used the gelator 3,5-bis-(5-hexylcarbamoyl-pentoxy)-benzoic acid hexyl ester (BHPB-6), which is a typical organogelator that is insoluble in water [26]. The molecular structure of the gelator is shown in Section 4.1.

LLC gels formed by gelling lyotropic nematic liquid crystals with LMWGs are to a certain extent the lyotropic equivalents to thermotropic liquid crystal elastomers (LCEs), which are of interest in fields such as biomimetics and soft actuation [27,28,29]. In LCEs, thermotropic liquid crystal monomers are covalently linked to a weakly cross-linked polymer network [27,30]. If the liquid crystal mesogens are aligned during the polymerization, the resulting material is stimuli-responsive, as triggers such as temperature or light can reversibly modify or destroy the liquid crystalline order. The change in the orientational order leads to a change in the macroscopic shape as the liquid crystal mesogen is coupled to the polymer chain [31,32,33]. For example, Ohm et al. synthesized highly shape-anisotropic, micrometer-sized particles from liquid crystalline elastomers [31]. Typical length scales are 200–500 µm. They observed that some particles contract while others expand during the nematic-to-isotropic transition, generating shape changes of 60% and 80%, respectively. The same principle should apply to lyotropic liquid crystal gels if the gel is formed in the macroscopically aligned LLC. Changes to the liquid crystalline order should induce a macroscopic (e.g., shape) change in the gel sample. Since LLCs are water-based, the orientational order of the LLC should be able to respond to additional triggers such as pH, vapor pressure, or salt concentration.

Another difference between LCEs and our LLC gels is the fact that the former are chemical gels synthesized via polymerization, while the latter are physical gels formed via the self-assembly of gelator molecules [23,34]. The formation of chemical gels is irreversible whereas physical gels can be destroyed and formed reversibly. In addition, the initial gel assembly is typically kinetically controlled and can reach a local energy minimum before it starts reorganizing over a longer timescale into a gel with different properties [7]; this process is referred to as gel aging.

**Figure 1:**
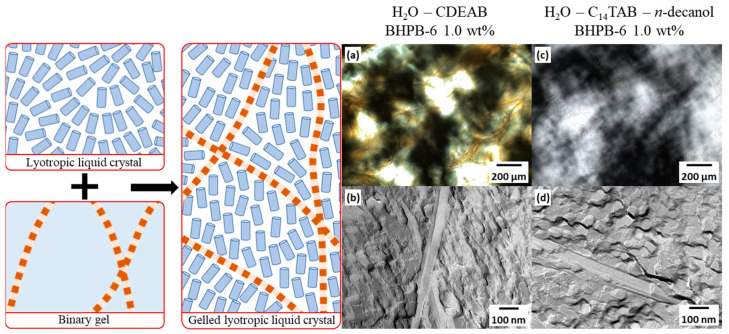
Schematic drawing of (**left**) a lyotropic liquid crystal and binary gel and (**right**) their combination, i.e., a gelled lyotropic liquid crystal. Blue cylinders represent the micelles and red strings the gel fibers. (**a**) POM and (**b**) FFEM images of the binary system H_2_O–CDEAB (g-N_C_(2)) gelled with 1.0 wt% BHPB-6. (**c**) POM and (**d**) FFEM images of the ternary system H_2_O–C_14_TAB–*n*-decanol (g-N_C_(3)) gelled with 1.0 wt% BHPB-6.

In this study, we gelled lyotropic nematic liquid crystals of binary and ternary systems using the LMWG BHPB-6 (Figure 1). We compared the gelling process of the binary and ternary systems over the course of 20 h for up to several weeks. The binary system consisted of the surfactant *N*,*N*-dimethyl-*N*-ethyl-1-hexadecylammonium bromide (CDEAB) and water, whereas the ternary system consisted of the surfactant *N*,*N*,*N*-trimethyl-*N*-tetradecylammonium bromide (C_14_TAB), the cosurfactant *n*-decanol, and water. The gelled calamitic nematic N_C_ phases of the binary and ternary systems will hereafter be referred to as g-N_C_(2) and g-N_C_(3), respectively.

## 2. Results and Discussion

### 2.1. Rheology

**Figure 2:**
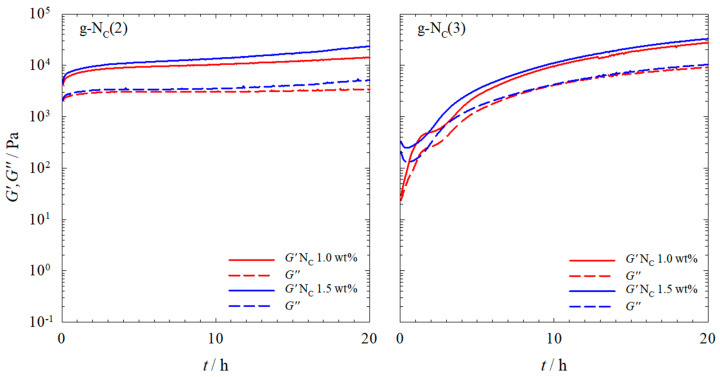
Storage modulus *G′* (straight line) and loss modulus *G″* (dashed line) of (**left**) the g-N_C_(2) system and (**right**) the g-N_C_(3) system measured at a constant temperature of *T* = 20 °C, shear strain of γ = 0.1%, and frequency of ω = 1 s^−1^. The N_C_ phases were gelled with 1.0 wt% (red) and 1.5 wt% (blue) BHPB-6.

We investigated the rheological properties of the gelled calamitic nematic N_C_ phases of (a) the binary system H_2_O–*N*,*N*-dimethyl-*N*-ethyl-1-hexadecylammonium bromide (CDEAB) and (b) the ternary system H_2_O–*N,N,N*-trimethyl-*N*-tetradecylammonium bromide (C_14_TAB)–*n*-decanol, both gelled with the gelator BHPB-6. The gelled calamitic nematic N_C_ phases of the binary and ternary systems will hereafter be referred to as g-N_C_(2) and g-N_C_(3), respectively. The gelation process of the samples was followed over the course of 20 h (Figure 2).

**Figure 3:**
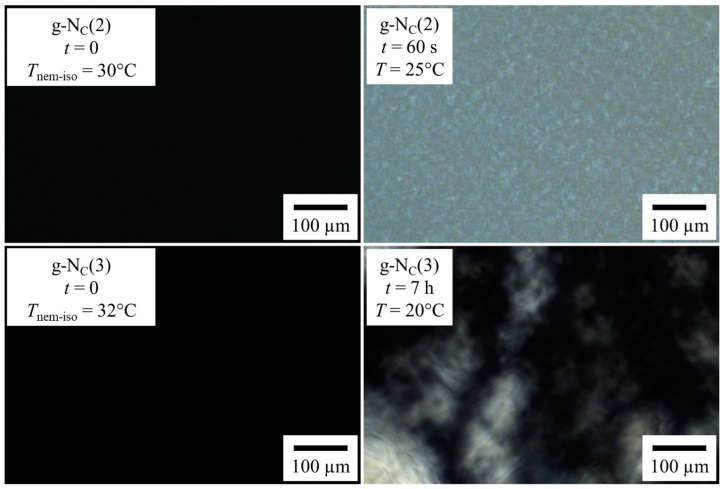
POM images between crossed polarizers of (**top**) the g-N_C_(2) system and (**bottom**) the g-N_C_(3) system recorded (**left**) shortly (*t* = 0) after reaching the phase transition temperatures, which are *T*_nem-iso_ = 30 °C for g-Nc(2) and *T*_nem-iso_ = 32 °C for g-Nc(3) and (**right**) after the formation of the N_C_ phase.

For the g-N_C_(2) samples gelled with 1.0 and 1.5 wt% BHPB-6, one observes *G′* and *G″* values of around 10^4^ and 2 · 10^3^ Pa, respectively, after 1 h, which increased only slightly over the course of 20 h. For the g-N_C_(3) samples gelled with 1.0 and 1.5 wt% BHPB-6, however, the *G′* and *G″* values increased by three orders of magnitude for the gel gelled with 1.0 wt% BHPB-6 and by two orders of magnitude for the gel gelled with 1.5 wt% BHPB-6. Moreover, the final *G′* and *G″* values were slightly higher compared to those of the g-N_C_(2) samples. These results showed that (a) the gelling process of the g-N_C_(2) system was much quicker compared to that of the g-N_C_(3) system, and (b) that the latter system formed slightly stronger gels after 20 h. Additionally, for the g-N_C_(3) system gelled with 1.0 wt% BHPB-6, a plateau was reached after two hours, before the *G′* and *G″* values continued to rise again. We assigned this plateau to a delay in the formation of the calamitic nematic N_C_ phase. This delay is clearly seen (Figure 3) if one heats the samples so that they become isotropic (*T* = 130 °C) and subsequently cools them to 20 °C, i.e., below their phase transition temperatures which are *T*_nem-iso_ = 30 °C for g-Nc(2) and *T*_nem-iso_ = 32 °C for g-Nc(3), as can be seen in the phase diagrams of the non-gelled systems (Section 4.1). It should be noted that the nematic phase formed (as usual in the absence of any external forces) as a non-aligned “polydomain” texture with the local director varying in space. We assumed that within each “domain”, the nematic order parameter reached its equilibrium value (typically 0.4 to 0.8) but have not measured this yet. Any measurement of the nematic order parameter (birefringence, X-ray diffraction, etc.) requires monodomain samples with the local directors macroscopically aligned into a single direction (e.g., by a magnetic field), and this is the subject of a follow-up study.

**Figure 4:**
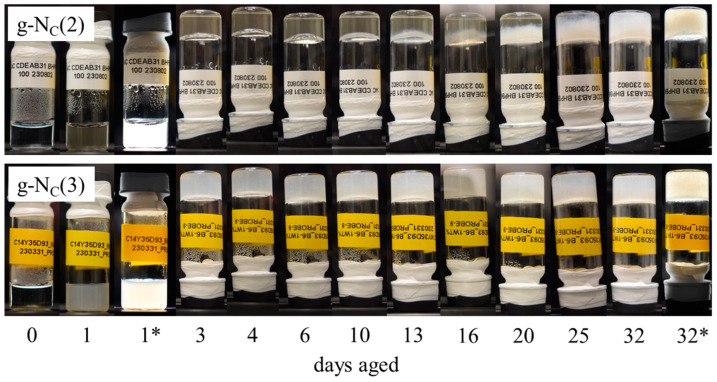
Gel aging of the g-N_C_(2) system (top) and the g-N_C_(3) system (bottom) gelled with 1.0 wt% BHPB-6. Images of the samples after 1 day and 32 days between crossed polarizers (*) were added to show the existence of the birefringent nematic N_C_ phase during the aging process.

In Figure 3, one sees that the nematic N_C_ phase forms within one minute in the g-N_C_(2) system, whereas in the g-N_C_(3) system, it takes some hours. We assigned this longer N_C_ phase formation time in the g-N_C_(3) system to the inclusion of *n*-decanol in the micelles, which was obviously kinetically hindered. The first appearance of the N_C_ phase could take one to three hours, with the full formation of the N_C_ phase taking up to 10 h. For both systems, a final plateau seemed to be reached after 20 h (Figure 2) with the samples gelled with 1.5 wt% having slightly higher *G′* and *G″* values than those gelled with 1.0 wt%. This would imply that the full gelation process took place within the first 20 h. However, further visual changes, predominantly in the g-N_C_(2) system, suggest that the gelation process was not finished after 20 h (Figure 4).

Both the g-N_C_(2) and g-N_C_(3) systems gelled with 1.0 wt% BHPB-6 were clear directly after preparation. It can be seen in Figure 4 (top) that after ~10 days, a turbid layer appeared for the g-N_C_(2) system, with the entire process taking a further 22 days for the sample to fully turn turbid. On the other hand, the g-N_C_(3) system started to become turbid already after 3–5 h and turned fully turbid within ~24 h (not shown here). The turbidity did not disappear over the course of 32 days as seen in Figure 4 (bottom). This observation was in line with gel aging processes seen in different systems [35,36,37], where the visual and physical properties of a gelled sample changed over the course of days and up to weeks. The g-N_C_(3) samples always turned turbid within 24 h, whereas the same process took days to start and weeks to finish for the g-N_C_(2) samples. In the case of the g-N_C_(3) system, the increasing turbidity was obviously connected to an increasing gel strength in the first 20 h. Whether this was also the case for the g-N_C_(2) system, which started getting turbid only after ~10 days, is an interesting question still to be answered.

**Figure 5:**
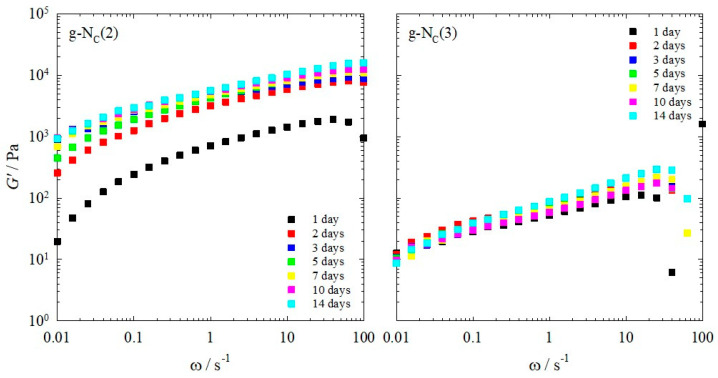
Rheology measurements obtained by oscillation frequency (ω)—sweeps at a constant temperature of *T* = 20 °C and constant shear strain of γ = 0.1% as a function of the aging time. The storage moduli *G′* for the nematic phases of (**left**) the g-N_C_(2) system and (**right**) the g-N_C_(3) system gelled with 1.0 wt% BHPB-6 are shown. For clarity, the loss moduli *G″* of the samples are not shown. Each measurement took ~3 h.

To study whether the *G′* and *G″* values of the g-N_C_(2) system changed with increasing turbidity, samples aged for significantly longer than 20 h needed to be measured. As it was unfeasible to keep the same sample within the rheometer for such a long time (both for practical reasons and evaporation effects), we chose to gel the samples outside of the rheometer. The gels were aged in glass vials, and after a given number of days, the samples were placed in the rheometer and measured for 3 h. The results are shown in Figure 5.

Looking at Figure 5, one sees that the *G′* values measured for the g-N_C_(2) system and the g-N_C_(3) system, respectively, differed significantly. The *G′* values of the g-N_C_(2) system were up to two orders of magnitude higher compared to those of the g-N_C_(3) system over the entire frequency range. Additionally, the *G′* values of the g-N_C_(3) system were much lower compared to the values shown in Figure 2, where the *G′* values of the g-N_C_(3) system surpassed those of the g-N_C_(2) system after 11 h for the samples gelled with 1.0 wt% BHPB-6. 

**Figure 6:**
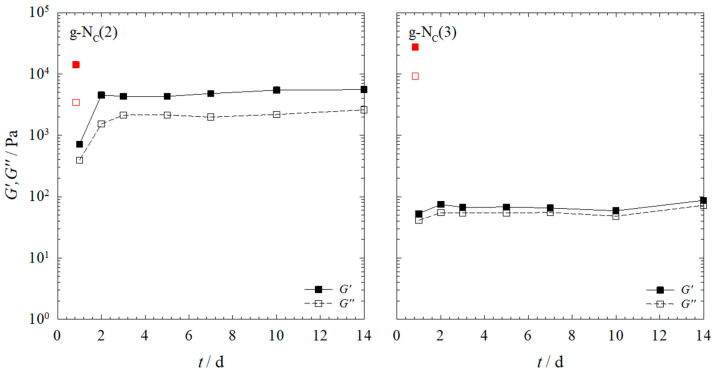
Storage modulus *G′* (filled boxes) and loss modulus *G″* (empty boxes) of the nematic N_C_ phases of (**left**) the g-N_C_(2) system and (**right**) the g-N_C_(3) system, aged for 1–14 days outside of the rheometer (black) and aged for 20 h within the rheometer (red). All samples were gelled with 1.0 wt% BHPB-6 and measured at a constant temperature of *T* = 20 °C, shear strain of γ = 0.1%, and frequency of ω = 1 s^−1^.

To clarify the unexpected changes observed over days and to compare them to the final values of *G′* and *G″* obtained after 20 h within the rheometer, the *G′* and *G″* values at a frequency of ω = 1 s^−1^ were plotted against the days each sample was aged. In addition, the *G′* and *G″* values of the samples aged 20 h within the rheometer are shown (red data points). We recall that the samples were kept outside the rheometer for aging and were measured after a given number of days (Figure 6).

**Figure 7:**
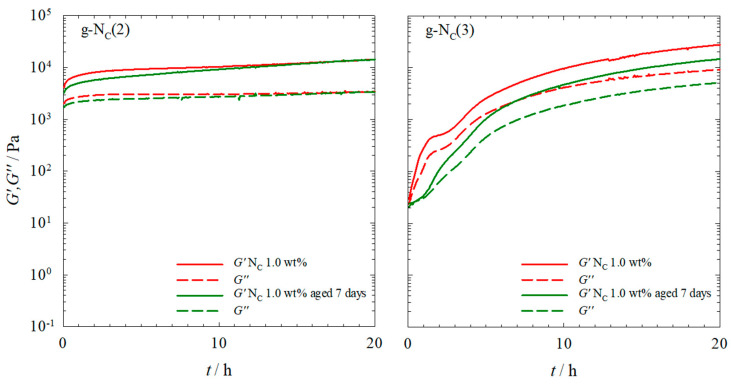
Storage modulus *G′* (straight line) and loss modulus *G″* (dashed line) of (**left**) the g-N_C_(2) system and (**right**) the g-N_C_(3) system recorded at a constant temperature of *T* = 20 °C, shear strain of γ = 0.1%, and frequency of ω = 1 s^−1^. The nematic phases were gelled with 1.0 wt% BHPB-6. The gelation took place within the rheometer for 20 h (red) or outside the rheometer for 7 days, and then samples were measured within the rheometer for 20 h (green).

Looking at Figure 6, one sees that, with the exception of the gelled g-N_C_(2) sample aged for 1 day, there was no change in the gel strength for both systems over the course of 14 days. Furthermore, comparing the *G′* and *G″* values of the samples gelled outside the rheometer (black) with those of the sample gelled within the rheometer after 20 h (red), one sees that the *G′* and *G″* values of the samples aged outside the rheometer were slightly lower in the case of the g-N_C_(2) system, but significantly lower in the case of the g-N_C_(3) system. Additionally, aging the samples for more than 1 day did not significantly increase the *G′* and *G″* values in either system. We assigned the difference in the *G′* and *G″* values of the samples gelled within and outside of the rheometer to a damaging of the gel structure when transferring the gelled sample from the glass vial to the rheometer. Obviously, the gel of the g-N_C_(2) system was less sensitive to mechanical disturbances during sample transfer. We hypothesize that this behavior was connected to the slow increase in turbidity, which, in turn, was an indication for the assembly of fibers. We further hypothesize that fiber assembly led to rigid rather than plastic/elastic gels, which were more prone to rupture. This is in line with visual observations that show that the clear g-N_C_(2) system was much more plastic/elastic compared to the turbid g-N_C_(3) system. As such, the removal from the vial and the placement in the rheometer had only a minor impact on the g-N_C_(2) system, while the more rigid gel of the turbid g-N_C_(3) system was destroyed during the removal from the vial and the placement in the rheometer. To confirm this, we compared two samples gelled in the rheometer with two samples gelled outside the rheometer (Figure 7). In the first case, the samples were placed in the rheometer plate as a sol at 90 °C and then measured for 20 h. In the second case, the samples were aged outside of the rheometer for 7 days and then placed on the plate and measured for 20 h.

Looking at Figure 7, one can observe that for the g-N_C_(2) system, there was only a slight difference in the *G′* and *G″* values of the two samples with their values converging after 20 h, whereas the g-N_C_(3) system showed a larger difference between the two differently prepared samples. Nevertheless, the results supported the assumption that the plasticity/elasticity of the g-N_C_(2) system made it less sensitive to the transfer process. Consequently, the *G′* and *G″* values were of the same order of magnitude, compared with those of the sample gelled within the rheometer. In the case of the g-N_C_(3) system, the rigid gel structure was destroyed during the transfer process, which was reflected in the fact that the *G′* and *G″* curves started at the values observed for the sample that was gelled in the rheometer. 

When comparing the *G′* and *G″* values of Figure 7 to those of Figure 6, which were measured ~3 h after placing the samples in the rheometer, one sees that they are in line with the samples gelled outside the rheometer after 3 h. For the g-N_C_(2) system, this time was sufficient for a nearly full gel formation. For the g-N_C_(3) system, however, 3 h was not sufficient to form the gel and the resulting *G′* and *G″* values were much lower compared to those of the samples gelled within the rheometer. The shape of the curves measured for the samples gelled outside of the rheometer was similar to that measured for the samples gelled within the rheometer. Note that the curves of the g-N_C_(2) system were nearly identical, whereas those of the g-N_C_(3) system were shifted to lower *G′* and *G″* values. Furthermore, the initial plateau of the g-N_C_(3) system was not present in the sample gelled outside of the rheometer. The cause of this plateau was assumed to be the delayed formation of the nematic N_C_ phase, which took hours. For the sample gelled outside of the rheometer, the N_C_ phase had enough time to be formed and thus no plateau was observed.

### 2.2. Freeze Fracture Electron Microscopy of the Gelled N_C_ Phases

It is shown in Figure 4 that the transparency of the gelled samples changed differently with time for the gelled Nc phase of the binary H_2_O–CDEAB and the ternary H_2_O–C_14_TAB–*n*-decanol systems, called g-N_C_(2) and g-N_C_(3), respectively. The g-N_C_(3) system became turbid within several hours after cooling the sample to room temperature, while for the g-N_C_(2) system, it required at least 10 days until the first turbid spots appeared and about 30 days for complete turbidity. 

**Figure 8:**
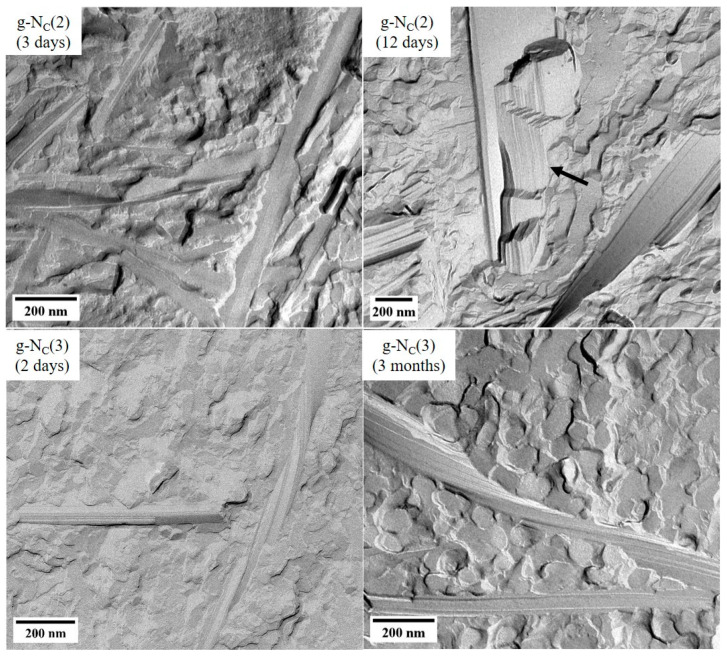
FFEM images of (**top**) the gelled Nc phase of the binary H_2_O–CDEAB (g-N_C_(2)) and (**bottom**) the ternary H_2_O–C_14_TAB–*n*-decanol (g-N_C_(3)) systems with different ages. The age of the samples is indicated in the pictures. All samples contained 1.0 wt% BHPB-6.

We used freeze fracture electron microscopy (FFEM) to determine whether the visually observed changes of the samples’ transparency with time could be assigned to a change in the BHPB-6 fibers’ structure. For this purpose, we prepared replicas of the transparent (aged for three days) and the completely turbid (aged for twelve days) g-N_C_(2) systems and examined them with a transmission electron microscope. The FFEM images of the two g-N_C_(2) samples are shown in Figure 8 (top), where significant differences between the transparent (left) and the turbid (right) sample can be seen immediately. For the transparent g-N_C_(2) system (Figure 8 top, left), we found single flat, non-twisted ribbons (or stacks of few ribbons) with a broad range of width (up to 125 nm). The average width of the ribbons was about 60 nm. Similar ribbons were already observed for binary gels consisting of cyclohexane and BHPB-6 [23,26]. For the turbid g-N_C_(2) system (Figure 8 top, right), we found very thick stacks of ribbons (black arrow in Figure 8) with a significantly larger width (up to ~660 nm) compared to the transparent g-N_C_(2) system. The average width of the ribbons in the turbid g-N_C_(2) sample was about 220 nm. It was not possible to determine the stack thickness for all the fibers, as it was not clearly visible for most fibers. In FFEM preparation, the fracture of the frozen sample most likely occurred at the weakest point, i.e., along the ribbon rather than through the ribbon stack. Nevertheless, a large number of stacks with a thickness between 100 and 850 nm were observed. Moreover, some stacked ribbons formed bundles over 1 µm thick. It should be mentioned that the twisting of some long, thin fibers was also observed. Obviously, it was unfavorable for the very thick ribbon stacks to twist.

Last but not least, FFEM images of two g-N_C_(3) samples (aged for two days and for three months) are also shown in Figure 8 (bottom). Both samples were turbid, and we did not find clear differences in the structures of the fibers. In both cases, we observed fibers consisting of several stacked ribbons, with a broad range of width (up to ~170 nm for the 2-day sample and up to ~200 nm for the 3-month sample). The average width of the ribbons was ~60 nm for the 2-day sample and ~80 nm for the 3-month sample. The stacks of ribbons in both the g-N_C_(3) samples were thicker than in the transparent g-N_C_(2) sample but not as thick as in the turbid g-N_C_(2) sample. Note that we observed a bit more of the thicker stacks of ribbons in the g-N_C_(3) sample aged for three months compared to that in the 2-day g-N_C_(3) sample. Some fibers in both the g-N_C_(3) samples were twisted, as one can see in Figure 8 (bottom).

Summing up, the FFEM images confirmed our assumption that the change in the sample transparency related to the structure of the BHPB-6 fibers in the g-N_C_(2) system. Here, fiber assembly happened over time (days), which made the samples turbid, and a certain fiber thickness was obtained. Up to 3 weeks were needed to turn the samples fully turbid. On the other hand, in the g-N_C_(3) sample, a turbid gel with thick stacks of ribbons already formed within 20 h, and according to the FFEM images, the structure of these BHPB-6 fibers did not change significantly thereafter. Comparing the obtained fibers with previous attempts to gel lyotropic liquid crystals using different gelators [12,13,16,17], one sees that they were significantly larger. Using the gelators DBS and 12-HOA, one obtains fibers with a width of 8–18 nm in the case of the former [16] and ~27 nm in the case of the latter [12]. With BHPB-6, however, much broader ribbons with widths of up to 660 nm in the case of the g-N_C_(2) system and 200 nm in the case of the g-N_C_(3) system were formed. Despite this large size, the nematic phase was undisturbed, contrary to attempts to gel the calamitic nematic phase using 12-HOA, where the presence of the gelator led to a phase transition into the lamellar phase [12]. This phase transition was due to the surface-active nature of the gelator 12-HOA and its tendency to act as a cosurfactant and be incorporated into the micelles [12]. On the contrary, the gelator BHPB-6 was not surface-active and the nematic phase was flexible enough to form around the thick fibers.

## 3. Conclusions

In this study, we investigated and compared the time evolution of the gelation process of two lyotropic nematic liquid crystal systems, namely, (a) the binary system H_2_O–CDEAB and (b) the ternary system H_2_O–C_14_TAB–*n*-decanol, each gelled with the gelator BHPB-6. The gelled binary and ternary systems are referred to as g-N_C_(2) and g-N_C_(3), respectively. We noted that the surfactants were quite similar from a structural point of view and that the main difference was the presence of a co-surfactant in the g-N_C_(3) system. Nevertheless, the gelation processes were quite different, showing that minor changes in intermolecular interactions can have an enormous effect on gel formation. 

We observed that the gelation processes of the g-N_C_(2) and g-N_C_(3) systems in the rheometer were different. While both systems reached similar *G′* and *G″* values after 20 h, the g-N_C_(2) system had high *G′* and *G″* values immediately, whereas those of the g-N_C_(3) system were very low at the beginning and subsequently increased over 20 h. Additionally, the *G′* and *G″* values of the g-N_C_(3) system gelled with 1 wt% BHPB-6 reached a plateau after ~3 h before rising again. We were able to assign this plateau to the formation of the nematic N_C_ phase using polarized optical microscopy (POM); while the inclusion of the cosurfactant *n*-decanol caused a delay in the formation of the N_C_ phase in the g-N_C_(3) system, which took from up to 3 to 10 h, the N_C_ phase of the g-N_C_(2) system formed immediately. Note that we did not find a plateau in the g-N_C_(3) system gelled with 1.5 wt% BHPB-6, indicating that the gel formation was influenced by the formation of the LLC phase at low gelator concentrations only. 

Apart from the differences observed in the rheological behavior of the g-N_C_(2) and g-N_C_(3) systems, we also found visual differences. In the case of the g-N_C_(2) system, the sample remained clear over the course of 20 h, while for the g-N_C_(3) system, the sample turned turbid over the same time frame. We correlated the change in turbidity to the rheological behavior of the samples: (a) The *G′* and *G″* values of the g-N_C_(2) system did not change significantly and the gel remained fully clear over the course of 20 h. (b) The *G′* and *G″* values of the g-N_C_(3) system changed significantly, and the gel changed from being clear to being turbid in the same timeframe. Observing g-N_C_(2) and g-N_C_(3) samples over the course of a month, we found that the g-N_C_(2) system also turned turbid after 10 days, showing that the gelation process was not over after 20 h but could extend to much longer timespans.

We used rheology to study g-N_C_(2) and g-N_C_(3) samples aged 1–14 days outside the rheometer. The resulting *G′* and *G″* values indicated that the transfer process to the rheometer plate destroyed the gel structure, with the g-N_C_(2) system being less affected than the g-N_C_(3) system. This was in line with visual observations that showed that the g-N_C_(2) system was more plastic/elastic, whereas the g-N_C_(3) system was more rigid and thus more affected during the transfer and preparation process. The obtained *G′* and *G″* values of both systems were independent of the aging time outside the rheometer, but they depended on the time spent inside the rheometer, which we assigned to the self-healing ability of the gels. Using FFEM, we showed that the size of the aggregates formed over long periods of time varied widely between the two systems. In the case of the g-N_C_(2) system, the stacking tendency of the fibers increased drastically, whereas for the g-N_C_(3) system, there was no clear visual difference between a sample aged 2 days and 3 months.

## 4. Materials and Methods

### 4.1. Materials and Sample Preparation

**Figure 9:**
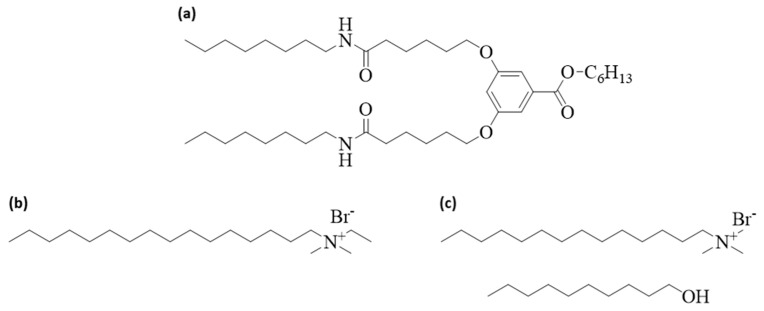
Molecular structures of (**a**) the gelator BHPB-6, (**b**) the surfactant *N*,*N*-dimethyl-N-ethyl-1-hexadecylammonium bromide (CDEAB), and (**c**) the surfactant *N*,*N*,*N*-trimethyl-*N*-tetradecyl-ammonium bromide (C_14_TAB) as well as the cosurfactant *n*-decanol.

For the gelation of the lyotropic liquid crystals, we used the gelator BHPB-6 (Figure 9a), synthesized as described in ref. [38]. The binary LLCs consisted of the surfactant *N*,*N*-dimethyl-N-ethyl-1-hexadecylammonium bromide (CDEAB, Merck KGaA, 98%) (Figure 9b) and bi-distilled water. The ternary LLCs consisted of the surfactant *N*,*N*,*N*-trimethyl-*N*-tetradecylammonium bromide (C_14_TAB, Thermo Scientific, 99%), the cosurfactant *n*-decanol (Merck KGaA, ≥99%) (Figure 9c), and bi-distilled water. All chemicals were used without further purification.

The gelator mass fraction η is given in wt% and calculated as follows:

(1) $$\eta_{BHPB-6}=\frac{m_{BHPB-6}}{m_{total\;sample}}\cdot 100\%.$$

The surfactant mass fraction µ of the binary CDEAB system is given in wt% as follows:

(2) $$\mu_{surfactant}=\frac{m_{surfactant}}{m_{surfactant}+m_{H_{2}O}}\cdot 100\%.$$

For the ternary C_14_TAB system, Equation (2) is modified to include the cosurfactant. Here, the surfactant mass fraction µ is calculated as follows:

(3) $$\mu_{surfactant}=\frac{m_{surfactant}+m_{cosurfactant}}{m_{surfactant}+m_{cosurfactant}+m_{H_{2}O}}\cdot 100\%.$$

The surfactant to cosurfactant ratio δ is kept constant and calculated as follows:

(4) $$\delta =\frac{m_{C_{14}TAB}}{m_{C_{14}TAB}+m_{n-decanol}}=0.93.$$

**Figure 10:**
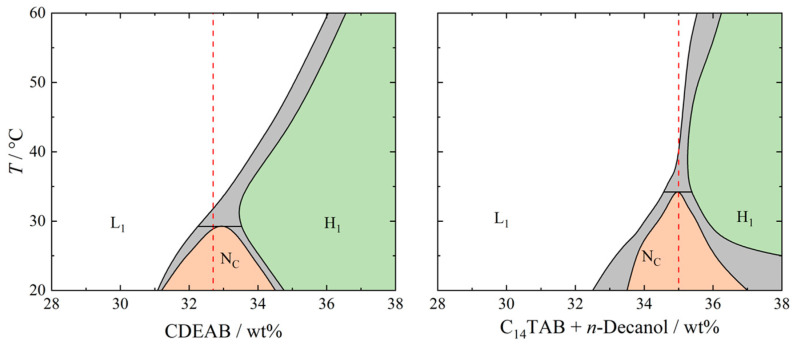
Phase diagrams of (**left**) the binary LLC system H_2_O–CDEAB and (**right**) the ternary LLC system H_2_O–C_14_TAB–*n*-decanol at a constant surfactant-to-cosurfactant ratio of δ = 0.93 [23]. The calamitic nematic N_C_ phase (orange), the micellar isotropic L_1_ phase (white), and the hexagonal H_1_ phase (green), as well as the two-phase regions (gray), are indicated. The red dashed lines highlight the concentrations that were used for the gelled N_C_ phases in the paper at hand.

The composition of the samples is shown in Table 1. The samples were prepared by weighing the compounds into a glass vial. The vial was closed using a screw-plug and further sealed using Teflon tape. The sample was then placed into a thermoshaker (Hettich MHR-23) and heated to 130 °C. Once this temperature was reached, the sample was shaken for an additional 10 min before being placed in storage at room temperature. This process was performed twice for each sample. The samples were then stored at room temperature for up to 14 days. Note that the sol–gel transition temperature was around 70 °C for the samples with 1.0 and 1.5 wt% BHPB-6. We heated the samples to up to 130 °C to fully homogenize them. During the cooling to room temperature, the gel fibers and the lyotropic nematic phase were formed. As can be seen in the phase diagrams (Figure 10), the isotropic-to-nematic phase transition temperature was *T*_nem-iso_ = 30 °C for the binary and *T*_nem-iso_ = 32 °C for ternary N_C_ phases.

### 4.2. Methods

Polarized optical microscopy (POM) was performed to verify the existence of the nematic phase in the gelled samples. The samples were filled into borosilicate flat capillaries (Electron Microscopy Sciences, Hatfield, USA) with a 0.300 mm wall thickness and dimensions of 0.30 mm × 3.0 mm. The capillaries were then flame-sealed, heated to 130 °C, and then left to cool to room temperature. The samples were left at room temperature for at least 1 day before being studied with a Leica DMLP polarizing microscope. The images were recorded using a Nikon D5300 (Nikon Corporation, Tokyo, Japan) camera.

Freeze fracture electron microscopy (FFEM) replicas of the samples were prepared using a Leica EM BAF060 freeze fracture and etching system. A small amount of the sample was placed on two copper grids and two copper plates with dimensions of 5.5 mm × 3.0 mm at room temperature. The grids were then assembled into a so-called sandwich and were subsequently frozen in liquid ethane cooled by liquid nitrogen. The samples were then transferred into liquid nitrogen before being fractured and transferred into the vacuum chamber of the BAF060 (stage temperature: −150 °C). The fractured samples were then shadowed by a layer of Pt/C (~2 nm) at an angle of 45° and covered by a layer of pure carbon (~20 nm) at 90°. The replicas were cleaned using ethanol and acetone, dried, and inspected using an EM10 transmission electron microscope (Carl Zeiss AG, Oberkochen, Germany) at 60 kV.

Oscillating shear rheometry was performed using a Physica MCR 501 (Anton Paar GmbH, Graz, Austria) rheometer with a plate–plate geometry (diameter of the upper plate: 25 mm) and a constant gap size of 1 mm. For all measurements, the temperature was set to *T* = 20 °C. The limit of the linear viscoelastic regime was determined by amplitude sweeps at a constant angular frequency of ω = 1 s^−1^ and an increasing shear strain (γ). Oscillation frequency sweeps were performed at a constant shear strain of γ = 0.1%, while varying the angular frequencies (ω) from 0.01 to 100 s^−1^. The samples used were aged for up to 14 days before being transferred from their vials onto the rheometer using a spatula. Long-term oscillation sweeps of the samples gelled within the rheometer were performed at a constant frequency of ω = 1 s^−1^ and constant shear strain of γ = 0.1% for 20 h. For the long-term oscillation sweeps, two different sample preparations were performed. In the first case, prior to the measurements, the samples were heated to 130 °C before being cooled to 90 °C and being poured onto the bottom plate of the rheometer. In the second case, the samples were gelled in a glass vial for 7 days before being transferred to the bottom plate of the rheometer using a spatula. The upper plate was afterwards immediately moved to the measuring position, and the sample was measured over the course of 20 h.

## Figures and Tables

**Table 1:** Composition of the samples.

Sample	BHPB-6/mg	CDEAB/mg	C_14_TAB/mg	H_2_O/mg	*n*-decanol/mg
Binary N_C_	-	330.0	-	670.0	-
g-N_C_(2) 1.0 wt% BHPB-6	10	326.7	-	663.3	-
g-N_C_(2) 1.5 wt% BHPB-6	15	325.0	-	660.0	-
Ternary N_C_	-	-	325.5	650.0	24.5
g-N_C_(3) 1.0 wt% BHPB-6	10	-	322.2	643.5	24.3
g-N_C_(3) 1.5 wt% BHPB-6	15	-	320.6	640.3	24.1

## Data Availability

Data will be made available on request.
